# Altered levels of BMD, PRL, BAP and TRACP-5b in male chronic patients with schizophrenia

**DOI:** 10.1038/s41598-020-70668-w

**Published:** 2020-08-12

**Authors:** Xiangdong Du, Fei Ye, Jin Li, Yaqin Zhao, Wenhuan Xiao, Xiaowei Tang, Xiaobin Zhang

**Affiliations:** 1 Institute of Mental Health, Suzhou Psychiatric Hospital, The Affiliated Guangji Hospital of Soochow University, Suzhou, 215137 Jiangsu People’s Republic of China; 2 Department of Psychiatry, Affiliated WuTaiShan Hospital of Medical College of Yangzhou University, Yangzhou, 225003 People’s Republic of China; 3 School of Mental Health, Jining Medical University, Jining, People’s Republic of China

**Keywords:** Biomarkers, Predictive markers, Prognostic markers

## Abstract

Bone mineral density (BMD) has been found to decrease in schizophrenia patients. We examined BMD and the levels of prolactin (PRL), bone alkaline phosphatase (BAP) and tartrate resistant acid phosphatase isoform 5b (TRACP-5b) in male chronic schizophrenia patients and compared them with healthy controls in a Chinese Han population, which has not been reported before. Male patients with chronic schizophrenia (SPs; n = 79) and healthy controls (HCs; n  = 56) were recruited. BMD and plasma PRL, BAP and TRACP-5b levels were measured and compared between the two groups. The SPs group was further divided into two subgroups: the elevated PRL group (PRL ≥ 25 ng/ml, EPRL; n  = 38) and the normal PRL group (PRL < 25 ng/ml, NPRL; n = 41) in accordance with PRL levels. The levels of BAP and TRACP-5b were measured using sandwich enzyme-linked immunosorbent assay (ELISA) while serum PRL was measured with an Access Immunoassay Analyzer. BMD was determined by quantitative computed tomography. BMD levels significantly decreased and serum PRL and TRACP-5b levels were significantly higher in male chronic schizophrenia patients. The EPRL group had remarkably lower BMD and BAP level and higher TRACP-5b levels compared with the NPRL group and HCs. Moreover, there was a negative correlation between BMD and TRACP-5b in the EPRL group. We found that BMD, BAP and TRACP-5b levels in the EPRL group were significantly different than HCs and the NPRL group. PRL levels in schizophrenia patients may be related to BMD and bone metabolism. Monitoring BMD and markers of bone metabolism in clinical practice may therefore be helpful to understand the bone health status of schizophrenia patients.

## Introduction

Osteoporosis is a metabolic bone disease characterized by reduction of bone mass and deterioration of skeletal tissues as well as abnormal bone metabolism^1^. Patients with osteoporosis have an increased bone fragility and are prone to fractures^2^. Osteoporosis can endanger human health and affect the quality of life^3^. Adolescence is a critical period of bone accumulation, and the peak bone mass during this period is influenced by genetics, length of adolescence, and light exposure and other factors^1,2,4^. Although genetically influenced, BMD is also influenced by various other factors including age, alcohol intake, smoking, nutrition, drug use, deficiency of vitamin D and various diseases^1,2,5–8^. Research has found that the risk of osteoporosis and femoral neck fracture in schizophrenia is higher than that of the general population^3,9,10^. In addition to lack of physical activity, smoking and drinking alcohol, poor nutrition and lack of sunshine, one of the primary reasons that schizophrenia patients are more likely to develop osteoporosis is long-term use of antipsychotics^11,12^.

Previous studies showed that use of antipsychotics has been associated with hyperprolactinemia (HPRL) and that PRL abnormality can affect metabolic and reproductive functions and endocrine systems in vivo^13,14^. HPRL influences bone metabolism by inhibiting the hypothalamus-pituitary–gonadal axis^11,15^ and by directly enhancing bone turnover^16,17^. However, there is inconsistency among studies investigating the correlation between elevated PRL resulting from antipsychotic treatment and bone metabolism in schizophrenia patients^11,18^.

Bone turnover markers can provide information about the status of bone formation and resorption. In clinical studies, these markers can reflect unremitting bone growth, predict fracture risk, and even monitor the treatment effect of osteoporosis^19,20^. Bone alkaline phosphatase (BAP), one of the most widely used bone formation markers, presents in osteoblast plasma membranes and is easily measured in serum or plasma. Tartrate resistant acid phosphatase isoform 5b (TRACP-5b), found in the ruffled border of osteoclasts, is characteristic of osteoclastic activity^20,21^. Bone turnover markers are altered prior to changes in BMD and can be detected in peripheral blood.

BMD is measured using a two-dimensional projection technique to detect the average concentration of minerals in the bone, primarily calcium. The most widely used method for measuring BMD is dual-energy X-ray absorption^1^. Commonly used measurement sites are the lumbar vertebrae and proximal femur^1^. Other techniques for measuring BMD include single-energy X-ray absorption, peripheral dual-energy X-ray absorptiometry, two-photon absorption, quantitative computed tomography (QCT) and quantitative sonication^2,22^. Among these methods, QCT can accurately measure the bone volume per cubic centimeter, which can more accurately indicate the bone mass, although a drawback of this technique is cost.

In addition, previous studies have found that the prevalence of osteoporosis is significantly different between men and women^23,24^. The pathophysiological mechanism and risk factors of bone mineral density reduction in male schizophrenia may be different from female schizophrenia^25,26^. Moreover, research of BMD in male schizophrenia is not sufficient.

Hence, we hypothesized that bone turnover markers and BMD may be changed in male chronic schizophrenia patients and increased PRL levels of the patients can contributed to their BMD reduction. Specifically, we investigated the levels of PRL, BAP, TRACP-5b and BMD to explore bone health in these patients. In addition, we attempted to explore whether there was a difference in BMD and bone transformation markers levels between different PRL levels groups.

## Materials and methods

### Subjects

Seventy-nine male patients with chronic schizophrenia from Affiliated WuTaiShan Hospital of Medical College of Yangzhou University of China were recruited for this study as the schizophrenia patient group (SPs). All patients enrolled in the study were determined by the Structured Clinical Interview for the Diagnostic and Statistical Manual of Mental Disorders, 5th edition (DSM-V), Patient Version (SCID-P) and were hospitalized for more than 5 years. The SCID and the positive and negative syndrome scale (PANSS) were conducted by the two experienced clinical psychiatrists separately (i.e. inter-rater correlation coefficient > 0.8) following the instructions and questions in the manual to identify the disorder in the SPs group, and to exclude any mental illness in the HCs group. All assessments were recorded before laboratory measurements were taken.

Exclusion criteria included patients with comorbidities including diabetes, dementia, mental retardation and alcohol or drug abuse/dependence; use of bone-specific treatment or other drugs that affected bone density, such as glucocorticoids; and being in a wheelchair or bedridden for an extended period.

All patients in the study were on a regular single oral antipsychotic treatment regime lasting 5 years or longer. Antipsychotics included chlorpromazine (n  = 13), risperidone (n  = 9), sulpiride (n  = 11), perphenazine (n  = 20), haloperidol (n  = 8) and clozapine (n = 18). Finally, the drug doses of each drug were converted into the equivalent dose of chlorpromazine.

Meanwhile, we enlisted 56 healthy male volunteers (HCs) from the local area in Yangzhou. All volunteers were in good health and had no mental illness history as assessed by DSM-5 Axis I psychiatric diagnosis.

The study was approved by the Ethics Committee of Yangzhou WuTaiShan Hospital. All subject signed an informed consent after receiving a complete clarification of the study’s goal and processes. All methods were performed in accordance with the 1964 Helsinki declaration and its later amendments or comparable ethical standards.

### Serum BAP, TRACP-5b and PRL level measurements

After fasting overnight, venous blood was taken from subjects between 07:30 and 08:30 of the next day. All blood samples were collected in tubes without an anticoagulant and centrifuged at 3000 g for 15 min. Samples were separated, aliquoted and stored at − 70 °C until further use.

The levels of BAP in serum were determined with an enzyme immunoassay (BAP; Quidel, San Diego, CA, USA) according to the manufacturer’s instructions. And the lowest limit of detection was 0.7 U/L, and the intra- and inter-assay coefficients of variation were 2.4% and 3.1%, respectively. Serum TRACP-5b concentrations were measured using an enzyme-linked immunosorbent assay enzyme assay kit (Cat #8033, Quidel Corporation,) according to the manufacturer’s instructions. The lowest limit of detection was 0.2 U/L and the intra- and inter-assay coefficients of variation were 3.2% and < 3.6%, respectively. An automated microplate reader (Epoch; BioTek Instruments, Winooski, VT, USA) was used to scale the optical density of each well. Serum PRL levels were measured by specialized hospital technicians using an Access Immunoassay Analyzer (Beckman-Coulter Inc., USA). The lowest limit of detection was 0.25 ng/ml, and the intra- and inter-assay coefficients of variation were o.4% and o.5%, respectively.

Patients and control samples were analyzed together on the same plates and were detected in the same time. All samples were tested in duplicate by researchers blinded to the experimental groups. BAP and TRACP-5b serum concentrations are presented in U/L while PRL serum concentrations were presented in ng/ml.

### BMD measures

BMD was determined by QCT using a Toshiba Aquilion 64-row CT scanner at the same day of blood sampling. A 5-sample solid QCT body model (Mindwany) was placed under the waist and lumbar vertebrae of the examinees for a lumbar spine spiral scan. The collected information was then transmitted to QCT pro workstation (Mindwany) for data analysis and three-dimensional images were acquired. The detection site was L2–4 vertebral cancellous bone. The average values of the three lumbar vertebrae were determined to obtain the BMD value. All lumbar vertebrae QCT examinations were performed by the same radiologist, and the measured BMD values were compared with the normal peer reference values. The mean BMD < 80 mg/cm^3^ measured by lumbar spine QCT was defined as osteoporosis, 80–120 mg/cm^3^ was considered as reduced bone mass, and > 120 mg/cm^3^ was regarded as normal.

### Statistical analysis

SPSS 21.0 (IBM, Armonk, NY, USA) was used to analyze data. Chi-squared analysis was applied for calculations of categorical data. All data were expressed as Median (IQR). *P* < 0.05 was considered statistically significant. Independent sample t-tests and Mann–Whitney U test were used to compare the difference between the SPs group and HCs.

Clinically, plasma PRL levels higher than 25 ng/mL were defined as HPRL^27^. So, we divided the SPs group into the elevated PRL group (PRL ≥ 25 ng/ml, EPRL) and the normal PRL group (PRL < 25 ng/ml, NPRL) in line with the serum PRL concentrations. The difference among the three groups (including HCs) was then compared. The differences among the three groups were analyzed by one-way analysis of variance (ANOVA) and the Kruskal–Wallis test.

Finally, the Pearson’s correlation and Spearman’s correlation analysis were used to analyze the correlation between BMD and BAP, TRACP-5b, PRL, duration of illness, chlorpromazine equivalents. And the key influence factors of BMD in schizophrenia patients were identified using generalized linear regression analyses after controlling for potentially confounding variables.

## Results

### Demographic and study data in the SPs and HCs groups

No significant difference was found between the two groups in age (IQR 43–50 vs. IQR 43–48; *t*  = 1.496, *P*  = 0.137), education (IQR 8–11 vs. IQR 6.25–11.75; *t*  = 0.926, *P*  = 0.357), smoking status (smoker/nonsmokers, 49/30 vs. 34/22; χ^2^  = 0.024, *P*  = 0.877) or BMI (IQR 22–26 vs. IQR 22.1–25.98; *t*  = 0.455, *P*  = 0.650). Median chlorpromazine equivalents was 500 (IQR 400–569) mg/day. At the time of the investigation, the median duration of treatments was 24(IQR 19–29) years. The median age of onset was 22(IQR 20–24) years and duration of illness was 25(IQR 19–29) years; all patients were between 36 and 57 years old.

Although there was no difference in BAP levels (IQR 22.8–34 vs. IQR 24.43–35.25 U/L; Z  = −1.076, *P*  = 0.282), the levels of TRACP-5b (IQR 3.8–5.8 vs. IQR 2.83–4.98 U/L; t  = 3.122, *P*  = 0.002), PRL (IQR19.56–33.06 vs. IQR 7.07–16.71 ng/ml; Z  = -7.925, *P*  = 0.000) and BMD (IQR 88.7–130.4 vs. IQR 113.1–146.03 mg/cm^3^; Z  = −3.209, *P*  = 0.001) in the SPs group were remarkably different from those in HCs (*P* < 0.05).

### Demographic and study data in the EPRL, NPRL group and HCs

There was no significant difference among the three groups in age, education, smoking status, or BMI (*P* > 0.05). There was also no significant difference in age of onset, duration of illness and treatment, chlorpromazine equivalents, or PANSS subscales scores between the EPRL and NPRL groups (*P* > 0.05; Table 1).

**Table 1:** Demographics, clinical characteristics in the EPRL, NPRL group and HCs.

	EPRL (n = 38)	NPRL (n = 41)	HCs (n = 56)	F/t/Z	*P* value
Age (years)	45 (42.75, 49)	47 (44, 50)	44 (43, 48)	1.815	0.167a
Education(years)	9 (8, 11)	9 (8, 11)	9 (6.25, 11.75)	2.064	0.356b
Smoker/Nonsmokers	21/17	28/13	34/22	1.438	0.487c
BMI (kg/m2)	25.4 (23.23, 26.45)	23.9 (22.65, 25.9)	23.9 (22.1, 25.98)	0.814	0.445a
BAP(U/L)	25.3 (18, 29.3)	30.8 (26.35, 38.05)	29.1 (24.43,35.25)	16.041	0.000b
TRACP-5b(U/L)	5.2 (4.68, 6.23)	4.2 (3.35, 5.35)	4 (2.83, 4.98)	12.032	0.000a
PRL(ng/ml)	33.21 (28.28, 47.37)	19.71 (17.67, 22.58)	10.49 (7.07 ,16.71)	90.154	0.000b
BMD(mg/cm3)	105.55 (80.05, 125.68)	119.2 (101.8, 135.85)	132.8 (113.1, 146.03)	16.280	0.000b
Age of onset (years)	22 (19.75, 24)	22 (20,24)		−0.222	0.824d
Duration of illness (years)	24 (19, 29.25)	26 (20.29.5)		−0.912	0.365e
Duration of treatment(years)	23.5 (19, 29)	24 (19, 29)		−0.747	0.457e
chlorpromazine equivalents	500 (400, 727.5)	500 (375, 650)		1.215	0.228e
PANSS positive subscale	11 (9, 14)	12 (9, 17)		−0.360	0.719d
PANSS negative subscale	17 (13.75, 20)	15 (14, 18)		−1.231	0.218d
PANSS general subscale	28.5 (23.5, 31)	27 (23.5, 29.5)		−0.772	0.440d
PANSS total score	55.5 (51.75, 64)	55 (50, 63)		0.171	0.864e

EPRL = the schizophrenia patients with elevated PRL levels; NPRL = the schizophrenia patients with normal PRL levels; HC = healthy controls; BMI = body mass index; BAP = Bone alkaline phosphatase; TRACP-5b = Tartrate Resistant Acid Phosphatase isoform 5b; PRL = prolactin; BMD = Bone mineral density; PANSS = Positive and Negative Syndrome Scale;

Data expressed as Median (IQR). Significant differences (P < 0.05) were marked in bold.

^a^One-way analysis of variance (ANOVA).

^b^Kruskal-Wallis one-way ANOVA(k samples).

^c^Chi-squared analysis.

^d^Mann-Whitney U test.

^e^Independent sample t-test.

**Figure 1:**
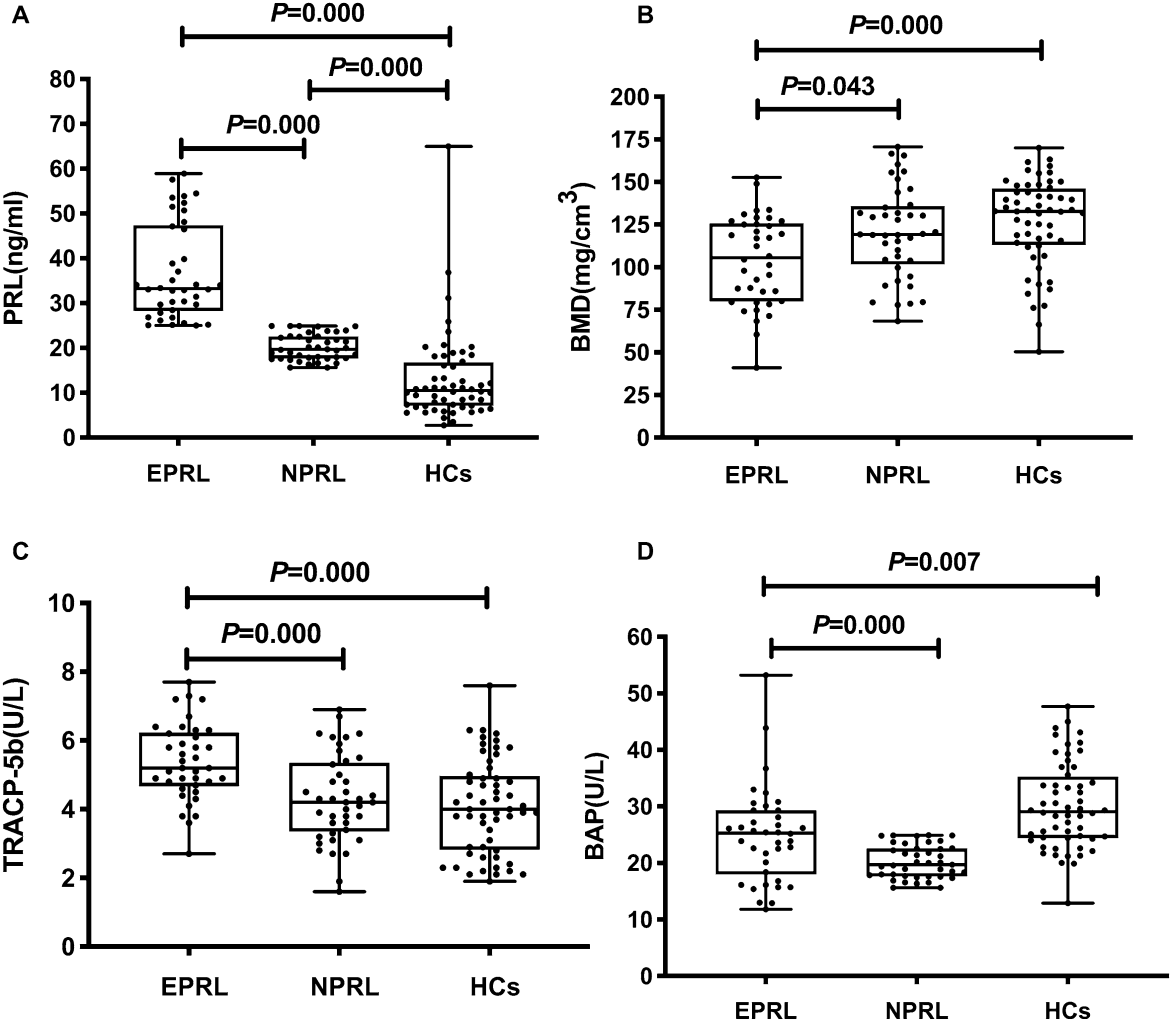
**(A)** Serum PRL levels in the schizophrenia patients with elevated PRL levels(EPRL), the schizophrenia patients with normal PRL levels(NPRL) and healthy controls(HCs); PRL = prolactin; **(B)** Serum BMD levels in EPRL, NPRL and HCs; BMD = Bone mineral density; **(C)** Serum TRACP-5b levels in EPRL, NPRL and HCs; TRACP-5b = Tartrate Resistant Acid Phosphatase isoform 5b; (**D)** Serum BAP levels in EPRL, NPRL and HCs; BAP = Bone alkaline phosphatase.

As expected, a remarkable difference among the three groups was found in relation to PRL levels according to the Kruskal–Wallis test (*P* = 0.000); Post hoc testing showed that there were significant differences between any two groups within the three ( EPRL VS NPRL, EPRL VS HCs, NPRL VS HCs, all *P* = 0.000; Fig. 1A).

The Kruskal–Wallis test proved that the differences in BMD in the three groups were significant (*P* = 0.000) and the BMD of patients in the EPRL group was sinificantly lower than that of patients in the NPRL group and in HCs (adjusted *P* = 0.043 and *P* = 0.000, respectively). However, there was no significant difference between the NPRL group and HCs (adjusted *P* = 0.451; Fig. 1B).

One-way ANOVA showed that there was a significant difference in TRACP-5b levels among the three groups (F = 12.032, df = 2, 132, *P* = 0.000). TRACP-5b levels in the EPRL group were remarkably higher than those in the NPRL group and in HCs (*P* = 0.000 and *P* = 0.000, respectively; Fig. 1C). Although TRACP-5b levels in the NPRL group were higher than those in HCs, the difference between the two groups was not significant (*P* = 0.400; Fig. 1C).

Following the Kruskal–Wallis test, BAP levels among the three groups also had a marked difference (*P* = 0.000). Compared with the other two groups, BAP levels in the EPRL group were significantly decreased (EPRL vs NPRL: adjusted *P* = 0.000; EPRL vs HCs: adjusted *P* = 0.007). Meanwhile BAP levels in the NPRL group were not markedly different from those in HCs (adjusted *P* = 0.791; Fig. 1D).

Duration of illness, chlorpromazine equivalents were not significantly related to BMD (all* P* > 0.05) in male schizophrenia patients. However, correlation analysis showed significant correlations between TRACP-5b (*r* =  − 0.300, *P* < 0.01), BAP (*r* = 0.301, *P* < 0.01), PRL (*r* =  − 0.431, *P* < 0.01) and BMD in men with schizophrenia. For the NPRL patients, there was no significant correlations between BMD and PRL, BAP , TRACP-5b, duration of illness, chlorpromazine equivalents (all *P* > 0.05). For the EPRL patients, no significant correlation was found between BAP (*r* = 0.213, *P* > 0.05) and chlorpromazine equivalents (*r* = 0.015, *P* > 0.05) with BMD. But TRACP-5b (*r* =  − 0.352, *P* < 0.05), PRL (*r* =  − 0.519, *P* < 0.01), duration of illness (*r* =  − 0.353, *P* < 0.05) were negatively correlated with BMD in male schizophrenia with elevated PRL levels.

Further generalized linear regression analyses was used to elucidate independent determinants of BMD. For male schizophrenia patients, generalized linear regression analyses used BMD as dependent variable, and TRACP-5b, PRL and BAP as covariates. We found that PRL (*β* =  − 0.976, *P* = 0.001) was an independent contributor to BMD. For the EPRL group’s patients, generalized linear regression analyses using BMD as dependent variable, and TRACP-5b , PRL and duration of illness as covariates revealed that TRACP-5b (*β* =  − 6.539, *P* = 0.036), PRL (*β* =  − 1.042, *P* = 0.006) were independent contributors to BMD.(Table 2).

**Table 2:** Generalized linear regression analyses with BMD as dependent variable.

	BMD					
	SPs			EPRL		
	*β*	SE	*P*	*β*	SE	*P*
BAP(U/L)	0.283	0.3208	0.378			
TRACP-5b(U/L)	− 2.307	2.2694	0.309	− 6.539	3.1228	**0.036**
PRL(ng/ml)	− 0.976	0.2901	**0.001**	− 1.042	0.3822	**0.006**

*β*, standardized coefficient; SE, standard error.

Significant differences (*P* < 0.05) were marked in bold.

## Discussion

In our study, the level of the BMD was significantly lower in the SPs group compared with HCs (Table 1, Fig. 1C). Our findings are consistent with previous studies of Shen, , et al.^10^, who reported that male schizophrenic subjects had a higher prevalence of decreased BMD compared with the healthy population in china. Cui, et al.^28^ also found that BMD decreased remarkably in Chinese patients with schizophrenia. However, Bergemann, et al. found that BMD in the lumbar and hip bones was normal in premenopausal women with schizophrenia compared with controls^29^. These discrepancies may be related to the gender of the study participants.

We also found that PRL levels in the SPs group were significantly higher than those in HCs (Table 1, Fig. 1B), which is in agreement with previous reports^15,30,31^. Elevated PRL may be associated with antipsychotic use in schizophrenia patients^14,15,32^, which results from the blocking effect of antipsychotic drugs on DA2 receptors in the anterior pituitary, but the effect of antipsychotic drugs on PRL levels is related to the mechanism of action of different kinds of drugs^33,34^.

After dividing the SPs group according PRL levels, we detected significant differences among the three groups (EPRL, NPRL and HCs). Compared with HCs, patients in the EPRL group had lower BMD. However, the difference of BMD between the patients in the NPRL group and HCs was not significant. Bulut et al.^12^ observed that BMD was lower in male outpatients who had used prolactin-raising (PR) antipsychotics as compared with the patients using prolactin-sparing (PS) antipsychotics (n = 19) and HCs. A meta-analysis revealed significantly lower BMD in schizophrenia patients than in HCs after pooling the results from 13 studies comparing BMD in schizophrenia patients and HC and from seven studies comparing BMD in patients receiving PR and PS^11^. In addition, the authors concluded that the BMD in schizophrenia patients taking PR was significantly lower than in those taking PS. However, a previous article^18^ reported that PRL levels were not directly related to insufficient bone mass, as assessed using the osteosono-assessment index ( used as an indicator of BMD ), among schizophrenia patients, but an association between PRL levels and low BMD was not excluded. Similarly, Lin, et al. found that PRL/hyperprolactinemia(HPRL) was not associated with BMD in women with schizophrenia^35^; they also found no significant association between PRL and BMD in 111 schizophrenia patients in a recent long-term follow-up study^36^.

The above findings were not in accord with our results. Several factors likely contributed to discrepancies in the previous and the current findings, including location and method of BMD measurement. Our results indicated that male chronic schizophrenia patients with high PRL levels were more likely to have reduced BMD, increased bone loss and even osteoporosis. Some studies suggested that HPRL may be associated with lower bone mass due to antipsychotic therapy in men with schizophrenia but not in women^23^. Two possible mechanisms are likely: one is the direct influence of elevated PRL levels increasing the bone turnover rate of patients^12,17^; and the other point is that increased PRL may lead to lower levels of estradiol and testosterone^23,26,37^, which can result in increased bone resorption and decreased BMD. In this regard, the relationship between PRL and BMD in schizophrenia patient needs further investigation.

It is worth noting that current studies have shown that reduced BMD in patients with schizophrenia is not only associated with elevated serum prolactin levels, but also with many other factors such as smoking, alcohol abuse, vitamin D deficiency and so on^1,2,4,10,11^. More studies without confounding influence of these variables are warranted.

We detected the serum levels of two bone metabolism markers (TRACP-5b and BAP) in all subjects and found that TRACP-5b levels were remarkably elevated in patients compared with those in HCs, while BAP levels between the two groups were not significantly different. These results indicated that bone resorption markedly increased in male chronic schizophrenia patients with significantly lower BMD than HCs. Therefore, these patients are more likely to develop osteoporosis than the general population. Of note, the difference in TRACP-5b and BAP levels was significant between the EPRL group and the NPRL group as well as HCs. Meanwhile these two markers of bone metabolism were similar in the NPRL group and in HCs.

Okita et al. ^38^found that no significant difference in serum TRACP-5b was found between SPs and HCs. In addition, some studies found no significant difference in serum BAP levels between chronic schizophrenia patients and HCs^39^. Bergemann et al. found elevated markers of bone formation and bone resorption (osteocalcin and urinary pyridine cross-linking) in 72 premenopausal women with schizophrenia who were received routine antipsychotic medication, and all had normal BMD in the lumbar and hip vertebrae^29^.

Again, the results of these studies are not completely consistent with our study, which may be related to the variety of BMD measurement sites selected in different studies, the different bone metabolism markers selected, and the different criteria for inclusion in the study subjects, and other factors. We found that BMD decreased and bone metabolism markers were altered in male chronic schizophrenia patients, especially in patients with elevated PRL levels. In animal studies, Seriwatanachai et al. ^17^ found that higher PRL levels may reduce bone formation and enhance bone resorption by increasing the expression of osteoclast differentiation factors and decreasing the expression of osteoprotective factor. PRL was also found to directly affect osteoblast differentiation and mineralization in vitro^16^. Several studies have proved that elevated PRL levels can also affect bone metabolism through sex hormones in schizophrenia^15,40^. The results of our study showed that bone formation was significantly reduced in the patients in the EPRL group and that bone resorption was notably increased. This particular finding is in agreement with results indicating that male schizophrenia patients with elevated PRL levels may have higher bone metabolism^23^.

Previous studies have shown lower BMD related to increased PRL, which were consistent with our results^15,23^. After regression analyses we found no significant relationship between BMD and bone metabolism markers in all chronic male schizophrenia patients. However, a significant correlation was found between TRACP-5b and BMD in male patients with higher PRL levels. Given the paucity of data, it is impossible to exactly determine if PRL and bone mass alterations are connected in schizophrenia patient, especially in the patients with higher PRL levels; therefore, more research is needed to determine if an actual interaction exists between the two. Importantly, the change in serum PRL levels and bone metabolism markers should be monitored closely by clinicians treating schizophrenia patients. Hence, PRL and bone metabolism markers should be examined to monitor the bone health status of schizophrenia patients, especially those who are on antipsychotic drug regimens for an extended period, in order to better prevent osteoporosis and fractures.

## Limitations

Several limitations in this study should be taken into account. First, all patients in the SPs group received antipsychotic medication; thus, the effects of different drugs on BMD and bone metabolism^41^ were not discussed in depth in our study. Further studies are needed to inquire into possible discrepancies using different antipsychotics groups, especially first-episode drug-naïve patients with schizophrenia. Second, bone is a dynamic structure that is constantly undergoing remodeling. Osteoclasts, osteoblasts and bone cells act together to mediate bone resorption and bone formation, which in turn determines bone mass^1,2,8^. Here we only tested for BAP and TRACP-5b, which does not fully reflect bone metabolism in patients. BMD and other bone turnover marker levels in schizophrenia patients should be studied in the future. Third, as previous studies has reported, reduced BMD in schizophrenia is associated with several factors such as smoking, physical activity, and alcohol consumption or other substance abuse^1,2,4,10,11^. However, due to the limitations of our study, we did not collect these data , which deserve future investigations. Lastly, the current study only recruited male patients and therefore did not represent the entire population and cannot be used to determine the bone health status of female patients^25^.

## Conclusion

In conclusion, male chronic schizophrenia patients had significantly lower BMD and higher TRACP-5b and PRL levels than HCs. Notably, the EPRL group showed increased TRACP-5b levels and decreased BAP levels compared with the NPRL group and HCs. Taken together, we speculate that PRL and bone metabolism markers may be related to the underlying mechanisms of reduced BMD in male chronic schizophrenia patients, thus providing a useful viewpoint to monitor the bone health of male schizophrenia patients on long term antipsychotics treatment.

## References

[1] Lupsa BC, Insogna K (2015). Bone health and osteoporosis. Endocrinol. Metab. Clin. N. Am..

[2] Järvinen TLN, Michaëlsson K, Aspenberg P, Sievänen H (2015). Osteoporosis: the emperor has no clothes. J. Internal Med..

[3] Wade SW, Strader C, Fitzpatrick LA, Anthony MS, O’Malley CD (2014). Estimating prevalence of osteoporosis: examples from industrialized countries. Arch. Osteoporos..

[4] Ko CH (2015). Deteriorating effect on bone metabolism and microstructure by passive cigarette smoking through dual actions on osteoblast and osteoclast. Calcif. Tissue Int..

[5] Milena F (2012). Role of obesity, alcohol and smoking on bone health. Front. Biosci..

[6] Emkey GR, Epstein S (2014). Secondary osteoporosis: pathophysiology and diagnosis. Best Pact. Res. Clin. Endocrinol. Metab..

[7] Yoon V, Maalouf NM, Sakhaee K (2012). The effects of smoking on bone metabolism. Osteoporos. Int..

[8] Drake MT, Clarke BL, Lewiecki EM (2015). The pathophysiology and treatment of osteoporosis. Clin. Ther..

[9] Stubbs B (2015). Schizophrenia and the risk of fractures: a systematic review and comparative meta-analysis. Gen. Hosp. Psychiatry.

[10] Shen Y, Li Z, Huang Y, Yan J, Liang Y (2017). Prevalence and factors associated with decreased bone mineral density in young and middle-aged male schizophrenic patients. J. Osteoporos. Phys. Activity.

[11] Tseng PT (2015). Bone mineral density in schizophrenia: an update of current meta-analysis and literature review under guideline of PRISMA. Medicine.

[12] Bulut SHD (2016). The effect of antipsychotics on bone mineral density and sex hormones in male patients with schizophrenia. Psychiatria Danubina.

[13] Inder WJ, Castle D (2011). Antipsychotic-induced hyperprolactinaemia. Aust. N. Z. J. Psychiatry.

[14] Franch C, Medina G, Ortega MD, Calzada ME, Molina V (2016). Problems in long-term treatment with atypical antipsychotics: hyperprolactinemia. Eur. Psychiatry.

[15] Marc DH, Johan D, Brendon S (2016). Relationship between antipsychotic medication, serum prolactin levels and osteoporosis/osteoporotic fractures in patients with schizophrenia: a critical literature review. Expert Opin. Drug Saf..

[16] Seriwatanachai D, Krishnamra N, van Leeuwen JPTM (2009). Evidence for direct effects of prolactin on human osteoblasts: inhibition of cell growth and mineralization. J. Cell. Biochem..

[17] Seriwatanachai D (2008). Prolactin directly enhances bone turnover by raising osteoblast-expressed receptor activator of nuclear factor kappaB ligand/osteoprotegerin ratio. Bone.

[18] Sugawara N (2011). No association between bone mass and prolactin levels among patients with schizophrenia. Hum. Psychopharmacol. Clin. Exp..

[19] Vasikaran S (2011). Markers of bone turnover for the prediction of fracture risk and monitoring of osteoporosis treatment: a need for international reference standards. Osteoporos. Int..

[20] Wheater G, Elshahaly M, Tuck SP, Datta HK, van Laar JM (2013). The clinical utility of bone marker measurements in osteoporosis. J. Transl. Med..

[21] Cao Y, LiuI X, Xu H (2012). Utility of serum tartrate-resistant acid phosphatase isoform 5b, bone alkaline phosphatase and osteocalcin in osteoporotic fractures in Chinese patients. Clin. Lab..

[22] Gong B, Mandair GS, Wehrli FW, Morris MD (2014). Novel assessment tools for osteoporosis diagnosis and treatment. Curr. Osteoporos. Rep..

[23] Kinon BJ, Liu-Seifert H, Stauffer VL, Jacob J (2013). Bone loss associated with hyperprolactinemia in patients with schizophrenia: are there gender differences?. Clin. Schizophr. Relat. Psychos..

[24] Rozental TD, Shah J, Chacko AT, Zurakowski D (2010). Prevalence and predictors of osteoporosis risk in orthopaedic patients. Clin. Orthop. Relat. Res..

[25] Jhon M (2018). Gender-specific risk factors for low bone mineral density in patients taking antipsychotics for psychosis. Hum. Psychopharmacol Clin. Exp..

[26] Lin CH (2015). Sex-specific factors for bone density in patients with schizophrenia. Int. Clin. Psychopharmacol..

[27] Melmed S (2011). Diagnosis and treatment of hyperprolactinemia: an endocrine society clinical practice guideline. J. Clin. Endocrinol. Metab..

[28] Cui J (2018). Prevalence, risk factors and clinical characteristics of osteoporosis in Chinese inpatients with schizophrenia. Schizophr. Res..

[29] Bergemann N, Parzer P, Mundt C, Auler B (2008). High bone turnover but normal bone mineral density in women suffering from schizophrenia. Psychol. Med..

[30] Grigg J (2017). Antipsychotic-induced hyperprolactinemia: synthesis of world-wide guidelines and integrated recommendations for assessment, management and future research. Psychopharmacology.

[31] Wang M (2014). Effects of antipsychotics on bone mineral density and prolactin levels in patients with schizophrenia: a 12-month prospective study. Hum. Psychopharmacol.: Clin. Exp..

[32] Mittal S, Prasad S, Ghosh A (2018). Antipsychotic-induced hyperprolactinaemia: case studies and review. Postgrad. Med. J..

[33] Haddad PM, Wieck A (2004). Antipsychotic-induced hyperprolactinaemia mechanisms. Clin. Features Manag. Drugs.

[34] Du X, Hill R (2019). Hypothalamic-pituitary-gonadal axis dysfunction: an innate pathophysiology of schizophrenia?. Gen. Comp. Endocrinol..

[35] Lin C-H (2012). Clozapine protects bone mineral density in female patients with schizophrenia. Int. J. Neuropsychopharmacol..

[36] Lin C-H, Lin C-Y, Wang H-S, Lane H-Y (2019). Long-term use of clozapine is protective for bone density in patients with schizophrenia. Sci. Rep..

[37] Crews MPK, Howes OD (2012). Is antipsychotic treatment linked to low bone mineral density and osteoporosis? A review of the evidence and the clinical implications. Hum. Psychopharmacol..

[38] Okita K (2014). Second-generation antipsychotics and bone turnover in schizophrenia. Schizophr. Res..

[39] Herran A (2000). Altered biochemical bone remodelling markers in schizophrenia. Eur. Psychiatry.

[40] Naidoo U, Goff DC, Klibanski A (2003). Hyperprolactinemia and bone mineral density: the potential impact of antipsychotic agents. Psychoneuroendocrinology.

[41] Wu H (2013). Osteoporosis associated with antipsychotic treatment in schizophrenia. Int. J. Endocrinol..

